# Tumoricidal Efficacy of Artesunate-Eluting Microsphere: Differential Role of Bax/Bak in Orchestration of Cell Death Pathways

**DOI:** 10.34133/bmr.0217

**Published:** 2025-06-10

**Authors:** Sarah Helmueller, Sanghee Lee, Xinxin Song, Dong-Hyun Kim, Yong J. Lee

**Affiliations:** ^1^ Department of Biomedical Sciences, Cedars-Sinai Medical Center, Los Angeles, CA 90048, USA.; ^2^ Department of Radiology, Feinberg School of Medicine, Northwestern University, Chicago, IL, USA.; ^3^ Department of Surgery, UT Southwestern Medical Center, Dallas, TX 75390, USA.; ^4^ Robert H. Lurie Comprehensive Cancer Center, Northwestern University, Chicago, IL, USA.; ^5^ Department of Biomedical Engineering, University of Illinois at Chicago, Chicago, IL, USA.; ^6^ Department of Biomedical Engineering, McCormick School of Engineering, Northwestern University, Evanston, IL, USA.

## Abstract

Artesunate (ART), an antimalarial drug, has been identified as a ferroptotic agent, inducing the generation of reactive oxygen species (ROS) and lipid peroxidation, which, in turn, activate endoplasmic reticulum (ER) stress responses and promote mitochondrial-dependent apoptosis. In our previous studies, we demonstrated that ART enhances tumor necrosis factor-related apoptosis-inducing ligand (TRAIL)-induced apoptosis through crosstalk between the ER stress-mediated signal pathway and the Bid–Bax mitochondrial apoptotic cascade. To further explore the mechanisms underlying ferroptotic–apoptotic crosstalk and evaluate the potential of intra-arterial drug-eluting microspheres for targeted tumor therapy, we developed artesunate-eluting microspheres (ART-EMs) and investigated the tumoricidal efficacy of ART-EMs combined with TRAIL. Our findings reveal that the combined ART-EMs with TRAIL (AT) treatment synergistically enhances cancer cell death. Specifically, we observed increased apoptosis in HCT116 and BxPC-3 cell lines, accompanied by notable morphological changes and enhanced cytotoxicity. Importantly, our results demonstrate that the pro-apoptotic proteins Bid and Bax play essential roles in driving synergistic apoptosis during AT treatment. Furthermore, the contrasting apoptotic responses between AT treatment and the chemotherapeutic agent mitomycin C’s dependence on p53–Bak-associated pathways underscore the differential activation of intrinsic apoptosis pathways across cancer cell lines. This study provides deeper insight into the roles of Bak and Bax in orchestrating apoptosis, offering potential strategies for more effective cancer treatments.

## Introduction

Historically, chemotherapy agents have been one of the few treatments that can prevent cancer tumor regression and promote cancer eradication from patients [1]. However, patients’ drug resistance overtime as well as the biologics’ potential impact on normal healthy cells create barriers to fully successful cancer treatment [2]. In order to improve upon monotherapy anticancer agents, combinatorial, targeted treatments have been studied as a way to have a synergistic–cytotoxic effect on tumor tissues while also limiting systemic toxicity [3,4].

Tumor necrosis factor (TNF)-related apoptosis-inducing ligand (TRAIL), a death receptor binding ligand, has been a highly studied drug because of its ability to selectively target cancer cells and not most normal cells [5,6]. TRAIL belongs to the TNF family of cytokines [5–7], and it extrinsically binds to death receptor 4 and 5 (DR4 and DR5), forming the death-inducing signaling complex (DISC) [8]. From our previous studies, we know that the formation of the DISC induces the caspase-dependent apoptosis pathway, which then intrinsically induces the Bid–Bax mitochondrial-dependent apoptosis pathway [7,9]. Caspase-8 activates executioner caspase-3 as well as cleaves the pro-apoptotic Bcl-2 protein Bid (BH3-interacting domain death agonist), forming tBid (truncated Bid), which translocates to the mitochondria. At the mitochondrial outer membrane (MOM), tBid initiates oligomerization of Bax (cytoplasmic Bcl-2-associated X protein) and Bak (Bcl-2 homologous antagonist killer) [7,9,10]. The Bax/Bak complex permeabilizes the MOM, allowing the release of cytochrome c into the cytosol, and subsequent activation of more caspases, culminating in apoptosis [7,9,11,12]. Previous experiments in vivo have shown that a combined treatment of TRAIL with a ferroptotic agent, such as anti-malarial agent, artesunate (ART), synergistically enhances apoptosis in cancer cell lines via the crosstalk between the ferroptotic agent-induced endoplasmic reticulum (ER) stress pathway and the intrinsic Bid–Bax mitochondrial-dependent apoptosis pathway [7,9,13,14].

Another difficulty in current cancer therapeutics is drug efficacy to target tumors [5]. One technology being used to overcome barriers to targeted delivery is the intra-arterial injection of microspheres [15–17]. This therapy is based on localized tumor targeting using hepatic intra-arterial infusion (HIAI) therapy and trans-arterial chemoembolization (TACE), where after injection the microspheres accumulate in the tumor blood vessels, blocking blood flow to the tissue and depriving the cancer cells of nutrients and inducing hypoxia [17,18]. Since we previously observed that hypoxia and glucose deprivation augment TRAIL-induced apoptosis [19,20], artesunate-eluting microsphere (ART-EM)-induced TACE would promote tumoricidal efficacy of TRAIL. Alongside HIAI and TACE, microspheres can be designed to be drug eluting, allowing the effective delivery of a drug and its slow release at the tumor site [15,16,21]. ART heightens the cell’s stress response by causing the production of reactive oxygen species (ROS) and increasing iron-dependent lipid peroxidation, culminating in ferroptosis [22]. As previously described, ART has been proven to enhance TRAIL-induced apoptosis through the crosstalk between the ferroptotic agent-induced ER stress signaling response pathway and the TRAIL-induced intrinsic Bid–Bax mitochondrial-dependent apoptosis pathway [7,9,13,14]. Therefore, a combinatorial treatment of ART-EMs and TRAIL (AT treatment) holds promise to first reduce the toxicity of therapeutics compared to singular drug treatments, as well as harness precise tumor targeting, HIAI, and TACE using ART-EMs.

Although other therapeutics, such as chemotherapy agents, can have effective cytotoxic effects on tumor tissues, it is necessary to understand both the effectiveness, cytotoxic, and, importantly, mechanistic differences between historically used chemotherapeutics versus the combinatorial treatment of a ferroptotic agent combined with an apoptotic agent as proposed in this study. A commonly used chemotherapeutic drug called mitomycin C (MMC) has been found effective in promoting DNA damage and apoptosis in bladder cancer cells, colorectal cancer cells, and metastatic colon cancer patients [23–25]. MMC, isolated from *Streptomyces caespitosus*, is an antibiotic that causes DNA alkylation and crosslinking, and is associated with ER stress-mediated apoptosis [24,26]. Similar to ART, MMC has been found to produce ROS and up-regulates stress signaling proteins and transcription factors in the ER [26]. As MMC is associated with S-phase DNA damage, it is thought to up-regulate other genes and proteins associated with this event, such as the ATM gene, which phosphorylates a transcription factor for cell cycle arrest and apoptosis called p53 protein [27–29].

Despite similar dependence on the ER stress-medicated apoptosis pathway, the separate therapeutic treatments of MMC versus ART-EM combined with TRAIL in cancer cells are an example of differential intrinsic pathways in cancer apoptosis and their dependency on pro-apoptotic Bcl-2 proteins to orchestrate cell death. In this study, we observed that MMC mediates apoptosis preferentially through the Bak- and p53-dependent apoptosis pathway, as compared to the Bid–Bax-mediated mitochondrial-dependent apoptosis pathway associated with ART-EMs and TRAIL combinatorial treatment.

## Materials and Methods

### Cell lines and cell culture conditions

Human colorectal carcinoma HCT116 cells and human pancreatic cancer BxPC-3 cells were previously obtained from the American Type Culture Collection (ATCC, Manassas, VA). Bid-deficient (Bid^−/−^) and Bid-deficient cells stably expressing Bid D60E or G94E vector (Bid^−/−^/D60E or Bid^−/−^/G94E) HCT116 cells were provided by X. Luo (University of Nebraska Medical Center, Omaha, NE). Bak-deficient (Bak^−/−^), Bax-deficient (Bax^−/−^), Bax/Bak double-knockout (Bax^−/−^ Bak^−/−^ DKO), and p53-deficient (p53^−/−^) HCT116 cells were kindly provided by B. Vogelstein (Johns Hopkins University, Baltimore, MD). Cell lines were maintained in media as listed: HCT116 cells in McCoy’s 5A and BxPC-3 cells in RPMI 1640 supplemented with 2 mM glutamine. All cell lines were maintained with 10% fetal bovine serum (FBS) and 1% penicillium streptomycin and incubated in a humidified atmosphere of 5% CO_2_ at 37 °C.

### Chemicals and reagents

For production of recombinant human TRAIL (rhTRAIL), a human TRAIL cDNA fragment (amino acids 114 to 281) obtained by reverse transcription polymerase chain reaction (RT-PCR) was cloned into a pET-23d plasmid (Novagen, Madison, WI), and His-tagged TRAIL protein was purified using the Qiagen express protein purification system (Qiagen, Valencia, CA). For the manufacturing of ART-EM, all reagents and solvents were obtained commercially and used without further purification. Poly(d,l-lactide-co-glycolide) (PLGA; lactide-glycolide ratio, 50:50), polyvinyl alcohol (PVA; 89,298 kDa, >99% hydrolyzed), dichloromethane, dimethyl sulfoxide, and methanol were purchased from Sigma-Aldrich (WI, USA). ART was purchased from TCI America (OR, USA). MMC was purchased from Cayman Chemical (Ann Arbor, MI).

### Manufacturing of ART-EM

ART-EM was prepared using the emulsion and solvent evaporation method. ART (10 mg) was dissolved in 200 μl of dimethyl sulfoxide and combined with 100 mg of PLGA and 3 mg of iron oxide nanoparticles (IONPs), which were dissolved in 4 ml of dichloromethane. The IONPs were synthesized using the thermal decomposition method, as described in our previous publication [30,31]. The solution was mixed vigorously using a vortex mixer for 1 min. This primary emulsion was then injected into an aqueous solution containing 2 wt % PVA to form an emulsion. The emulsification process was performed using a homogenizer at 1,500 revolutions per minute (RPM) for 5 min. The organic solvent in the emulsion was evaporated for 2 h at room temperature. ART-EMs were collected by centrifugation at 2,000 RPM for 5 min and washed 3 times with deionized water. The final solution was lyophilized and stored at −20 °C. The morphologic characteristics and size distribution of ART-EM were analyzed using an ECHO Revolve phase microscope (ECHO, CA, USA) and scanning electron microscopy (SEM; Hitachi S-4800; Hitachi, Tokyo, Japan). The loaded ART was dissolved in methanol (>99%) and quantified using a ultraviolet–visible (UV-vis) spectrophotometer (Synergy HT, BioTek, VT, USA). The loaded IONP was quantified using an inductively coupled plasma mass spectrometry (ICP-MS; Thermo iCAP Q). The loading contents of ART and IONP were calculated as follows: mass of ART and IONP/total mass of ART-EM × 100.

### Cell morphology study

Cells were treated with ART-EM and/or TRAIL and observed under an ECHO Revolve phase microscope (ECHO, San Diego, CA, USA) in the bright-field mode.

### Cell death and viability assay

Cell death was measured using the trypan blue exclusion assay to detect the cells’ plasma membrane integrity. To quantify the percentage of cell death, cells were trypsinized and stained with 0.4% trypan blue, followed by counting using the LUNA Automated Cell Counter (L10001, Logos BioSystem, Anyang, Gyeonggi-do, South Korea) according to the manufacturer’s instructions.

### Western blotting and antibodies

Immunoblotting was carried out as previously described [13]. The following antibodies were used in this study: anti-PARP-1 (#9532), anti-caspase-3 (#9664), anti-caspase-8 (#9746), anti-caspase-9 (#7237), anti-Bax (#2772), anti-Bak (#3814), anti-Bid (#2003), anti-BIM (#2819), and anti-p53 (#2524) (Cell Signaling Technology, Beverly, MA), and anti-actin, goat anti-rabbit immunoglobulin G (IgG)–horseradish peroxidase (HRP), and goat anti-mouse IgG-HRP (Santa Cruz Biotechnology, Santa Cruz, CA).

### siRNA transfection

Small interfering RNA (siRNA) was transfected into cells using Lipofectamine 3000 (Thermo Fisher Scientific) and the manufacturer’s protocol. SignalSilence Bim siRNA (#6461) was used (Cell Signaling Technology, Beverly, MA). Cells were treated with siRNA and Lipofectamine for 24 h, and then drug treatment was performed for another 24 h. Cells were then harvested and analyzed using immunoblotting.

### Combination index analysis

Combination index (CI) analysis was performed using CompuSyn software (ComboSyn Inc., Paramus, NJ, USA). The extent of antagonism/synergism was determined based on CI values. CI values above 1 suggest antagonism between the drugs, whereas CI values below 1 indicate synergy. CI values in the 0.9 to 1.10 range mainly indicate additive effects, those between 0.9 and 0.85 suggest slight synergy, those in the range of 0.7 to 0.3 indicate moderate synergy, and those less than 0.3 suggest strong synergy.

### Statistical analysis

Statistical analysis was performed using 1-way and 2-way analysis of variance (ANOVA) followed by Sidak’s or Tukey’s multiple comparisons test as indicated using GraphPad Prism 8 software. *P* values less than 0.05 were defined as statistically significant. *P* values are indicated as follows: *P < 0.05; **P < 0.01; ***P < 0.001.

## Results

### Preparation and characterization of ART-EMs

**Fig. 1.**
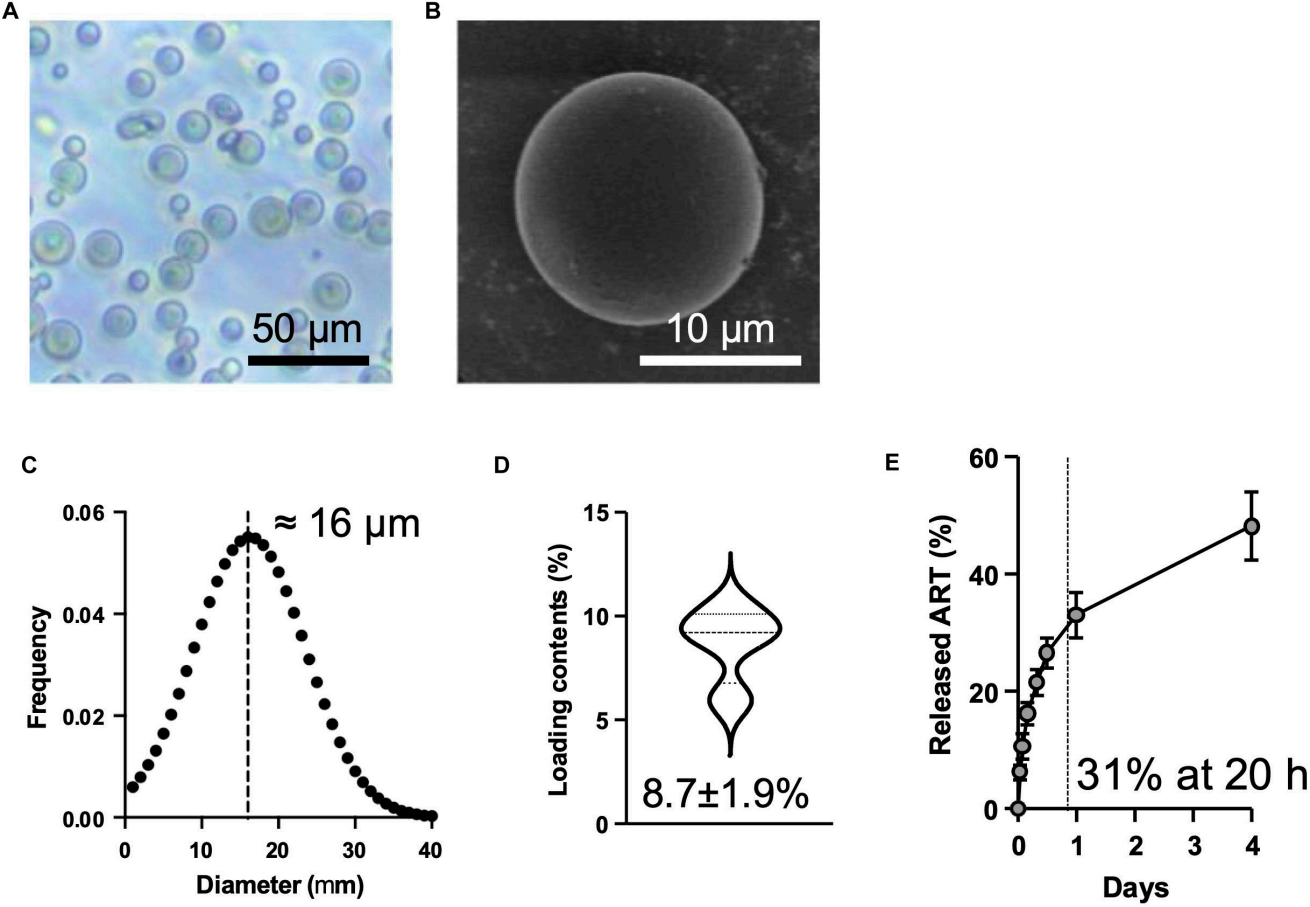
Characterization of ART-EMs. (A) Representative optical images of ART-EMs. Magnification: ×40. Scale bar, 50 μm. (B) SEM images showing the surface morphology of ART-EMs. Scale bar, 50 μm. (C) Size distribution of ART-EM, determined by measuring the diameter (*n* = 100). (D) Loading contents of ART (left *y* axis) and IONP (right *y* axis), quantified by UV-vis spectroscopy at 252 nm and ICP-MS, respectively (*n* = 4). (E) Accumulative release profile of ART from ART-EM over 4 d using UV-vis spectroscopy (*n* = 3).

Biodegradable ART-EM was prepared using a single emulsion method [32]. The embedded 20-nm cube-shaped IONPs exhibited contrast effects in magnetic resonance imaging (MRI), enabling ART-EM to facilitate real-time monitoring of its distribution and confirm the delivery accuracy to targeted tumors [16]. The spherical morphology of ART-EM was observed in optical microscope images (Fig. 1A). In SEM images, a well-formed ART-EM displayed a smooth and uniform surface (Fig. 1B). The mean hydrodynamic size of ART-EM was approximately 16 μm, determined by measuring the diameter of 100 representative ART-EMs observed in optical microscope images (Fig. 1C). The ART loading content was approximately 8.4 ± 1.9% of the total mass of ART-EM, and the IONP loading content was approximately 1.2 ± 0.1%, consistent with our previous findings for sorafenib-eluting microspheres (Fig. 1D) [30–33]. The release profile of ART from ART-EM was evaluated under physiological conditions. The cumulative release of ART was monitored over time to assess its release kinetics. The results demonstrated an initial burst release followed by a sustained release, with approximately 30.6% of the total ART content being released within the first 20 h. The subsequent release rate showed approximately 48.3% for 4 d (Fig. 1E).

### ART-EMs combined with TRAIL induces cytotoxicity in HCT116 and BxPC-3 cells

**Fig. 2.**
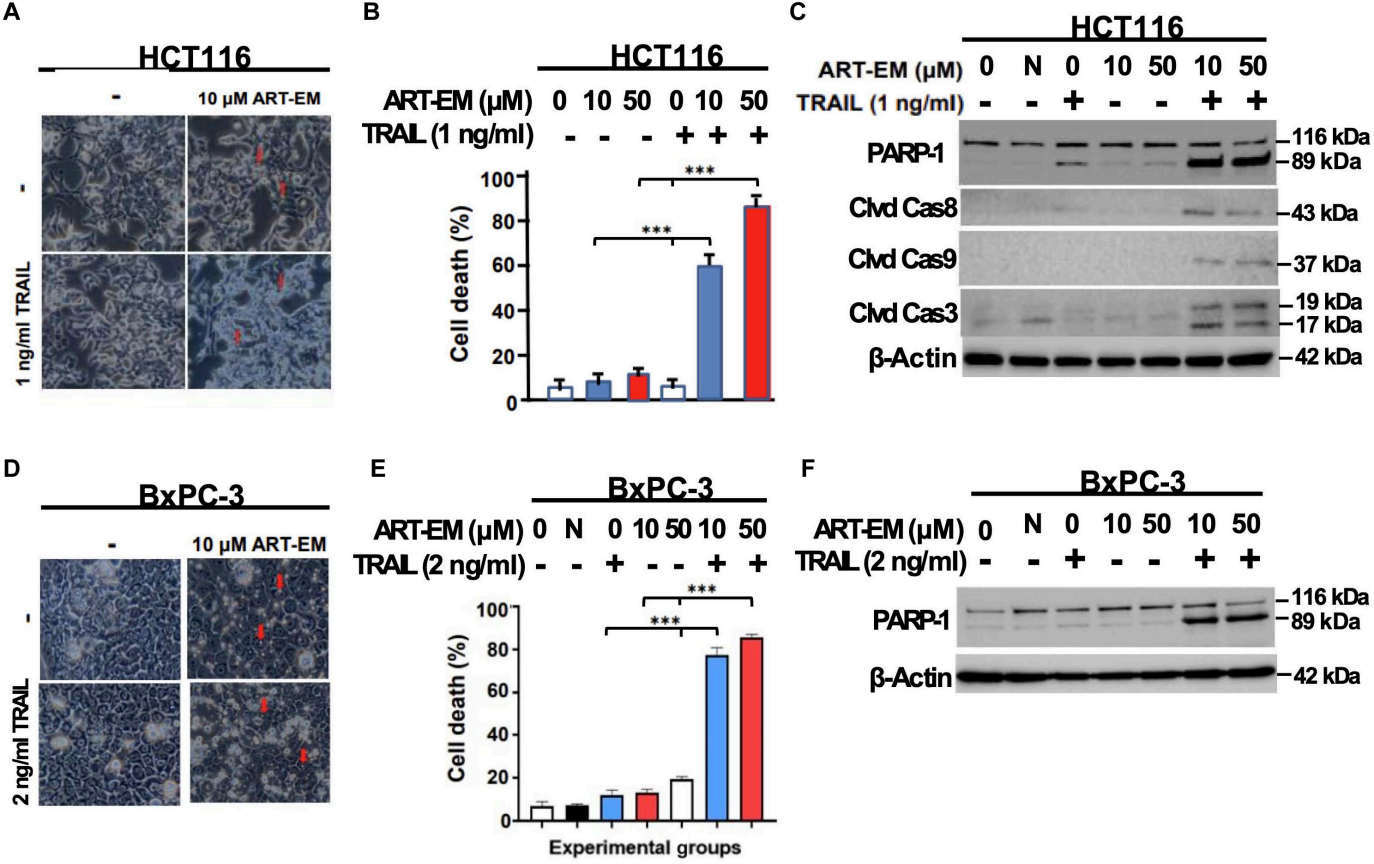
ART-EMs promotes TRAIL-induced apoptosis in human colorectal carcinoma HCT116 and human pancreatic adenocarcinoma BxPC-3 cells. Cells were treated with 10 or 50 μM ART-EM for 20 h and then treated with or without rhTRAIL (1 ng/ml for HCT116 or 2 ng/ml for BxPC-3 cells) for another 4 h. (A and D) After treatment, morphological features were analyzed under an ECHO Revolve microscope. Red arrows indicate the presence of ART-EMs. (B and E) Cell death was determined by a trypan blue exclusion assay. Error bars represent the mean ± SD from triplicate experiments, and CI analysis was used to assess the amount of synergy between combined drug doses. CI index: ***, strong synergy. N, negative control using 50 μM microspheres alone. (C and F) Whole-cell extracts were analyzed with an immunoblotting assay using indicated antibodies. N, negative control using 50 μM microspheres alone.

Our previous studies observed cytotoxicity of ART combined with TRAIL in a variety of cancer cell lines [7,9,13]. In this study, we employed ART-EMs with and without TRAIL to explore their combinatorial cytotoxic effect in HCT116 and BxPC-3 cell lines. Comparatively, dual treatment of HCT116 cells with ART-EMs and TRAIL (AT) shows enhanced cell detachment and membrane blebbing when compared to the control or single treatment (10 μM ART-EMs, 1 ng/ml TRAIL, 2 ng/ml TRAIL) samples (Fig. 2). These cell morphology changes indicate increased cytotoxicity in the AT-treated samples (Fig. 2A and D). To further investigate the synergistic cytotoxicity in cancer cell lines when treated with ART-EMs and TRAIL, we employed the trypan blue exclusion assay to assess cell survival and death rates (Fig. 2B and E). Our results demonstrate strong synergistic cytotoxicity based on CI values below 0.3 in the AT dual treatment compared to any single treatment type or untreated sample in both cell lines (Fig. 2B and E and TTable).

**Table.** Combination index for ART-EM and TRAIL

Combination therapy	Combination index
TRAIL (ng/ml)	ART-EM (μM)	HCT116
1	10	0.00297 ^a^
1	50	0.0000466 ^a^
TRAIL (ng/ml)	ART-EM (μM)	BxPC-3
2	10	0.000402 ^a^
2	50	0.0000736 ^a^

^a^ Strong synergy.

### ART-EMs and TRAIL induce synergistic cytotoxicity through apoptosis

Treating cancer cell lines with the ferroptotic agent, ART-EMs, and apoptotic agent TRAIL together enhances their cytotoxic effect. To uncover the mechanism behind ferroptotic agent and apoptopic agent synergy, we employed Western blotting techniques. Western blot analysis of HCT116 cell extracts and BxPC-3 cell extracts clearly shows enhanced PARP-1 cleavage, the hallmark of apoptosis, in samples treated with the combination of ART-EMs and TRAIL (Fig. 2C and F). The quantity of cleaved PARP-1 in the AT treatment is greater than that in TRAIL or ART-EMs alone (Fig. 2C and F). Cleaved caspase-8, cleaved caspase-9, and cleaved caspase-3 in the AT-treated samples also indicate caspase-8, caspase-9, and caspase-3 activation, which, again, marks apoptosis as the mechanism of cell death during AT treatment (Fig. 2C and F).

### ART-EMs and TRAIL promote apoptosis depending on the presence of functional Bid

**Fig. 3.**
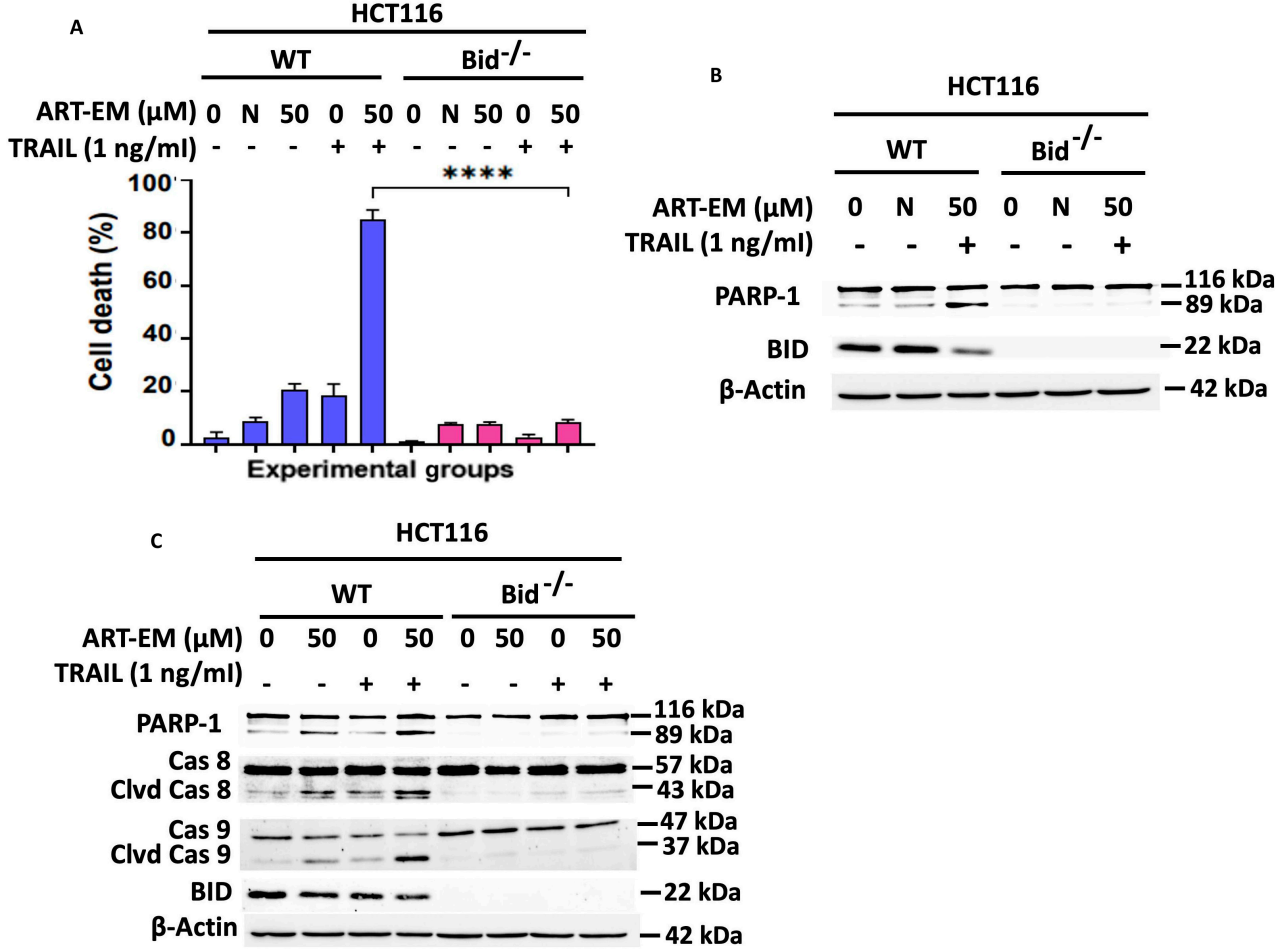
Role of Bid in the synergistic interaction between ART-EMs and TRAIL in HCT116 cells. HCT116 WT and Bid^−/−^ cells were treated with 50 μM ART-EM for 20 h and then treated with or without 1 ng/ml rhTRAIL for another 4 h. (A) After treatment, cell death was determined by a trypan blue exclusion assay. Error bars represent the mean ± SD from triplicate experiments. ****P = 0.0001. N, negative control using 50 μM microspheres alone. (B) Whole-cell extracts were analyzed with an immunoblotting assay using indicated antibodies. N, negative control using 50 μM microspheres alone. (C) Whole-cell extracts were analyzed with an immunoblotting assay using indicated antibodies.

To further understand the mechanism of synergistic cytotoxicity observed in the combined treatment using ART-EMs and TRAIL, we tested its dependence on the presence of functional Bid. As shown in our previous studies, functional Bid is required for synergistic crosstalk between the ferroptotic agent-induced ER stress pathway and the TRAIL-induced apoptosis pathway in samples treated with both ART and TRAIL [7]. To confirm if this mechanistic dependence on Bid holds consistent with ART-EMs and TRAIL combined, we used the trypan blue exclusion assay to assess cell death and the effect of Bid deficiency in cancer cell cytotoxicity. Results affirm that Bid plays a key role in the synergistic cytotoxicity of ART-EMs and TRAIL, as we observed little cell death in Bid^−/−^ cell lines, and over 80% cell death in wild-type (WT), AT-treated samples (Fig. 3A). The efficacy of the AT treatment was further validated using Western blot analysis, which shows suppression of PARP-1 cleavage and caspase-8 and caspase-9 cleavage in Bid-deficient cell lines (Fig. 3B and C).

**Fig. 4.**
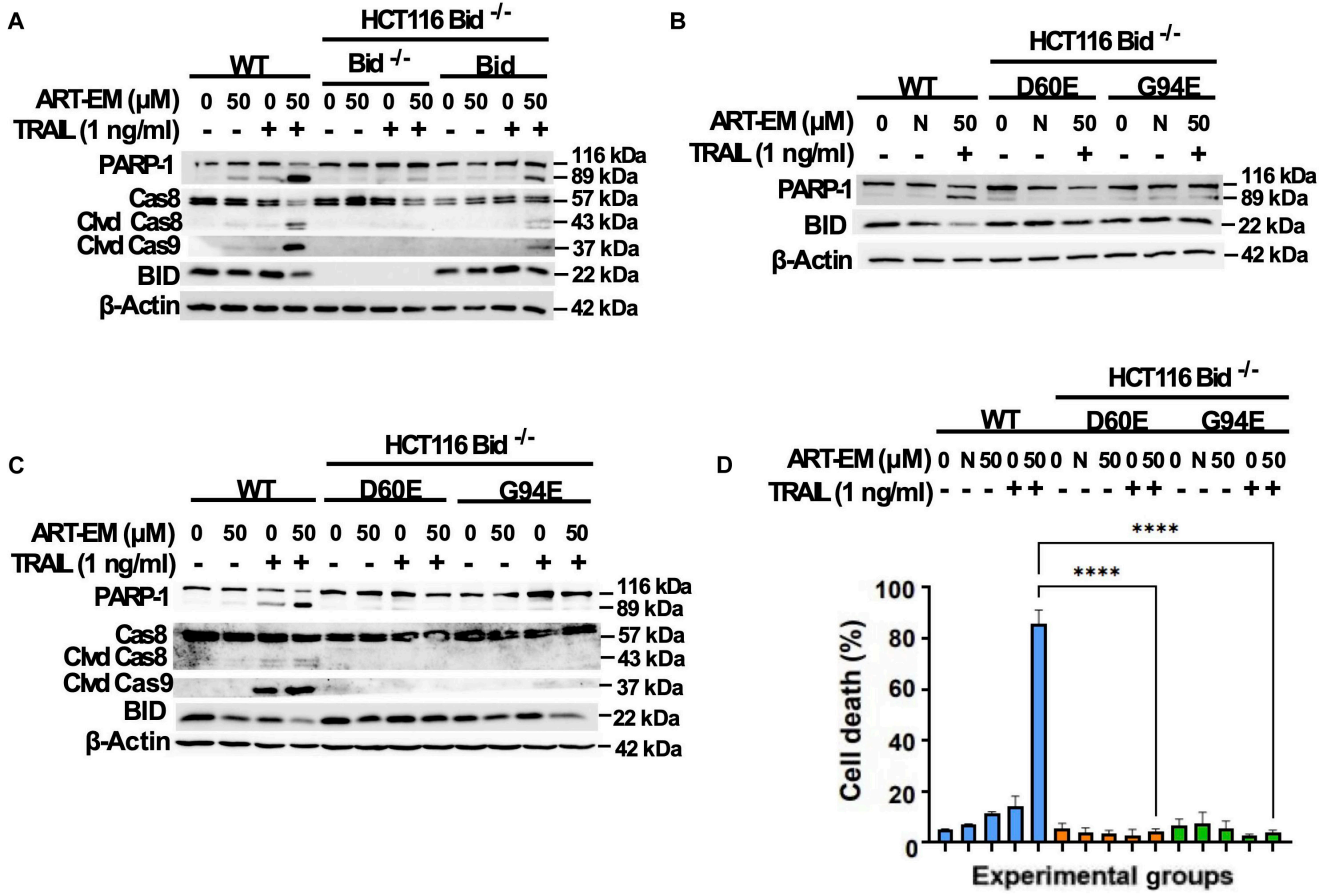
Functional Bid is required for the synergistic apoptosis in ART-EM-enhanced TRAIL-induced apoptosis. HCT116 WT, Bid^−/−^, Bid^−/−^/Bid, Bid^−/−^/D60E, and Bid^−/−^/G94E cells were treated with 50 μM ART-EM for 20 h and then treated with or without 1 ng/ml rhTRAIL for another 4 h. (A to C) Whole-cell extracts were analyzed with an immunoblotting assay using indicated antibodies. N, negative control using 50 μM microspheres alone. (D) After treatment, cell death was determined by a trypan blue exclusion assay. Error bars represent the mean ± SD from triplicate experiments. ****P = 0.0001. N, negative control using 50 μM microspheres alone.

In order to further confirm ART-EM and TRAIL’s substantial dependence on Bid as a key regulator in the crosstalk between ferroptosis and apoptosis signaling pathways, we treated Bid-deficient cell lines alongside the stable cell line variants restored with functional Bid, Bid D60E (mutation at Bid cleavage site), and Bid G94E (mutation in BH3 domain) [7]. Synergistic apoptosis was observed in WT and Bid-reconstituted Bid^−/−^ cells during the combinatorial treatment with ART-EMs and TRAIL, as shown through Western blot analysis and subsequential PARP-1 cleavage and caspase-8 and caspase-9 cleavages (Fig. 4A) [7]. Apoptosis was not observed in either Western blot data collected from Bid D60E or Bid G94E mutant cell line extracts, or in cell survival data, proving that fully functional Bid is paramount in orchestrating synergistic cytotoxicity during AT treatment (Fig. 4B and C).

### MMC induces Bid- and Bim-independent apoptosis

**Fig. 5.**
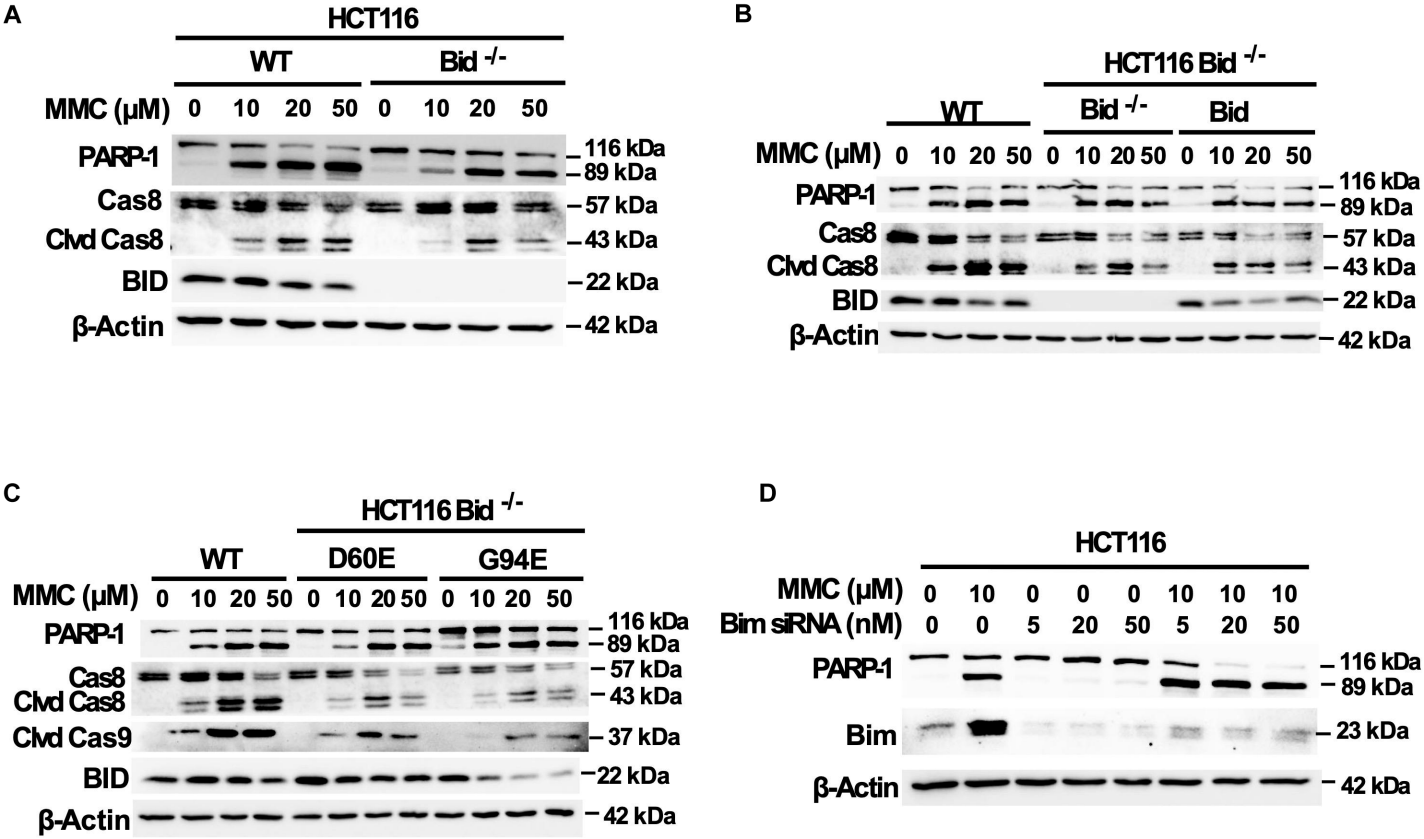
Bid is not required for MMC-induced cytotoxicity. HCT116 WT, Bid^−/−^, Bid^−/−^/Bid, Bid^−/−^/D60E, and Bid^−/−^/G94E cells were treated with 10, 20, or 50 μM MMC for 24 h. (A) Whole-cell extracts were analyzed with an immunoblotting assay using indicated antibodies to compare treatment effects in WT HCT116 cells and Bid^−/−^ cells. (B) Again, whole-cell extracts were analyzed with an immunoblotting assay using indicated antibodies to compare treatment effects in WT HCT116 cells, Bid^−/−^ cells, and Bid^−/−^/Bid cells, respectively. (C) To compare treatment effects in WT HCT116 cells, Bid^−/−^/D60E, and Bid^−/−^/G94E cells, whole-cell extracts were analyzed with an immunoblotting assay. (D) Before treatment with 10 μM MMC for 24 h, HC116 WT cells were transfected with Bim siRNA using Lipofectamine 3000 kit. Then, whole-cell extracts were analyzed with an immunoblotting assay using indicated antibodies.

With the aim to deduce whether the Bid-dependent signaling mechanism seen in the combinatorial treatment is unique to the synergistic cytotoxicity of ART-EMs combined with TRAIL, the next analysis looks at the mechanism of a chemotherapeutic, MMC. As proven above, the combinatorial treatment-induced ferroptotic–apoptotic crosstalk mechanism is dependent on Bid, which is a member of the family of BH3 domain proteins, a subclass of the Bcl-2 group [7,34]. Some studies show varied reliance on BH3 proteins (Bid, Bad, Bim, Bik, EGI-1, BMF, NOXA, BNIP3, and Beclin-1) as key regulators in chemotherapeutic-initiated apoptosis [34–36]. To assess this, we compared MMC-treated Bid-deficient cell extracts to Western blot results observed in ART-EMs and TRAIL-treated samples. Unlike in combinatorial ART-EMs and TRAIL-treated samples, 10, 20, and 50 μM MMC-treated HCT116 WT, Bid-deficient (Bid^−/−^), and Bid-reconstituted Bid-deficient, Bid D60E, Bid G94E cells all resulted in PARP-1 cleavage and caspase-8 cleavage, both signatures of apoptosis (Fig. 5). Interestingly, these results show that MMC’s cytotoxic effect is not dependent on functional Bid. However, it is still possible that MMC could be dependent on another BH3 domain protein, such as Bim [36]. The role of Bim in the MMC-induced apoptosis pathway was assessed using siRNA knockdown techniques; HCT116 WT cells were transfected with Bim siRNA and then treated with or without MMC. The successful transfection of Bim siRNA is confirmed by Western blotting results and the reduction of Bim protein in untreated, transfected samples as compared to the untreated, nontransfected control sample (Fig. 5D). Continually, these results exemplify MMC’s Bid-independent and Bim-independent apoptosis, as PARP-1 cleavage still occurred in MMC-treated, Bim siRNA-transfected samples (Fig. 5D). Overall, we see a significant mechanistic difference between the combinatorial treatment of ART-EMs and TRAIL versus an exemplar chemotherapeutic agent regarding their differential reliance on BH3 domain proteins, Bid and Bim.

### Bax orchestrates the crosstalk between ferroptotic and apoptotic cell death pathways

**Fig. 6.**
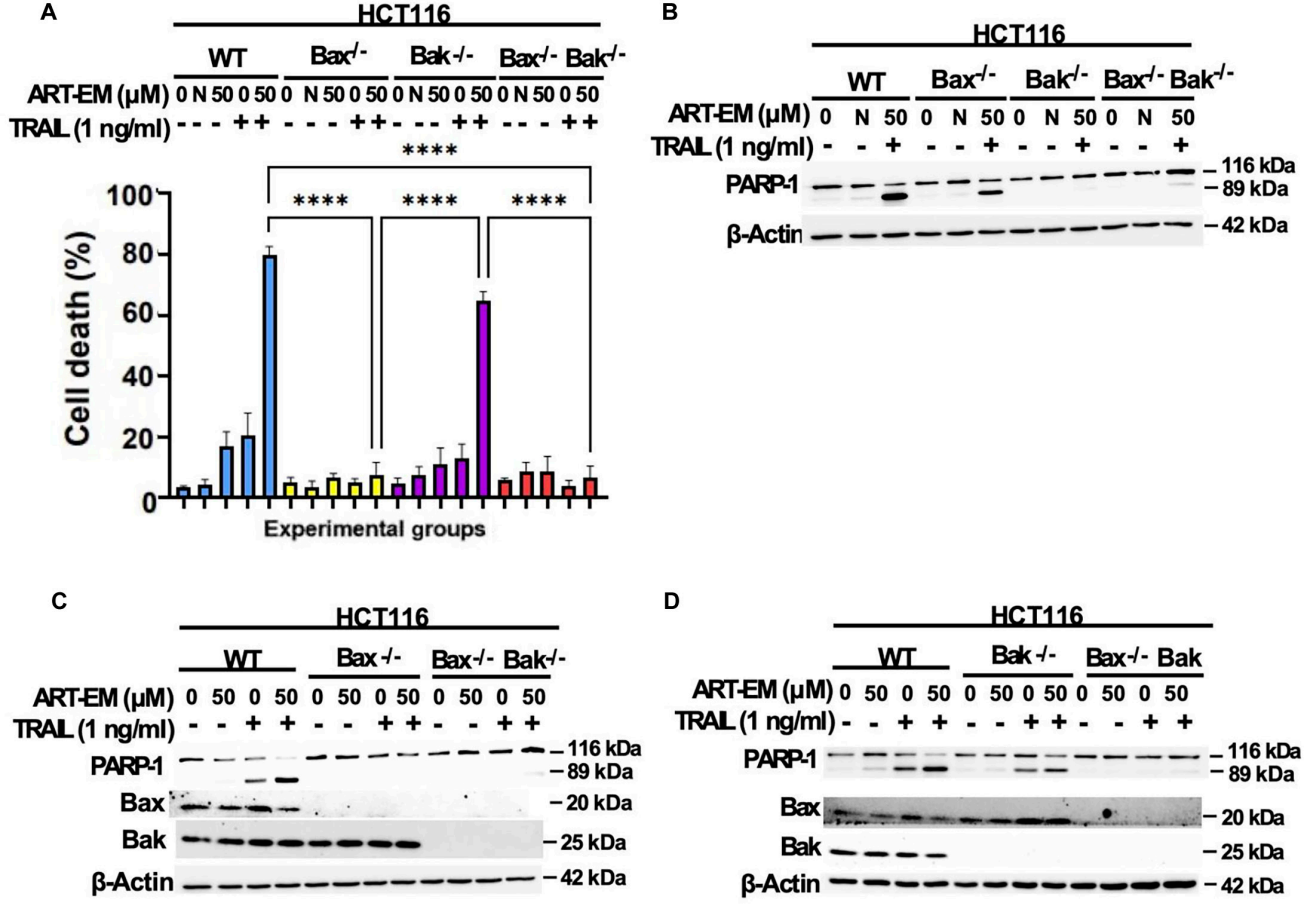
Role of Bax and Bak in ART-EM-enhanced TRAIL-induced apoptosis. HCT116 WT, Bak^−**/**−^, Bax^−**/**−^, and Bak^−/−^/Bax^−/−^-DKO cells were treated with 50 μM ART-EM for 20 h and then treated with or without 1 ng/ml rhTRAIL for another 4 h. (A) After treatment, cell death was determined by a trypan blue exclusion assay. Error bars represent the mean ± SD from triplicate experiments. ***P = 0.001; ****P = 0.0001. N, negative control using 50 μM microspheres alone. (B) Whole-cell extracts were analyzed with an immunoblotting assay using indicated antibodies. N, negative control using 50 μM microspheres alone. (C) Whole-cell extracts were analyzed with an immunoblotting assay using indicated antibodies to compare treatment effects in WT HCT116 cells, Bax^−/−^ HCT116 cells, and Bak^−/−^/Bax^−/−^-DKO HCT116 cells. (D) Whole-cell extracts were analyzed with an immunoblotting assay using indicated antibodies to compare treatment effects in WT HCT116 cells, Bak^−/−^ HCT116 cells, and Bak^−/−^/Bax^−/−^-DKO HCT116 cells.

The results above confirm that the BH3 domain protein Bid is important in ART-EMs and TRAIL combinatorial treatment in cancer cell lines. Another critical set of Bcl-2 proteins previously shown to have a significant role in the combinatorial AT treatment is the Bax–Bak complex, which oligomerizes via tBid in the intrinsic mitochondrial apoptosis signaling pathway and releases cytochrome c [7,9]. We previously reported that Bax specifically mediates the ART and TRAIL-induced crosstalk between ferroptosis and apoptosis, while Bak is shown to not be significant [9]. ART-EMs and TRAIL combinatorial-treated HCT116 cells confirmed these previous observations, with the trypan blue exclusion cell viability assay demonstrating that Bax-deficient and Bax–Bak DKO cell lines had no significant cytotoxicity, especially compared to the WT and Bak-deficient samples (Fig. 6A). In Western blot analysis, the lack of PARP-1 cleavage in Bax-deficient and Bax–Bak DKO cell lines further confirms that ART-EMs and TRAIL combinatorial treatment is mainly dependent on Bax, not Bak, as a signaling molecule in the crosstalk between the ferroptotic-induced ER stress signaling pathway and the intrinsic mitochondrial apoptosis pathway (Fig. 6B to D). Bax and Bak antibody staining confirms the absence of Bax and/or Bak in the various KO cell lines (Fig. 6B to D).

### MMC is preferably reliant on Bak-dependent apoptosis

**Fig. 7.**
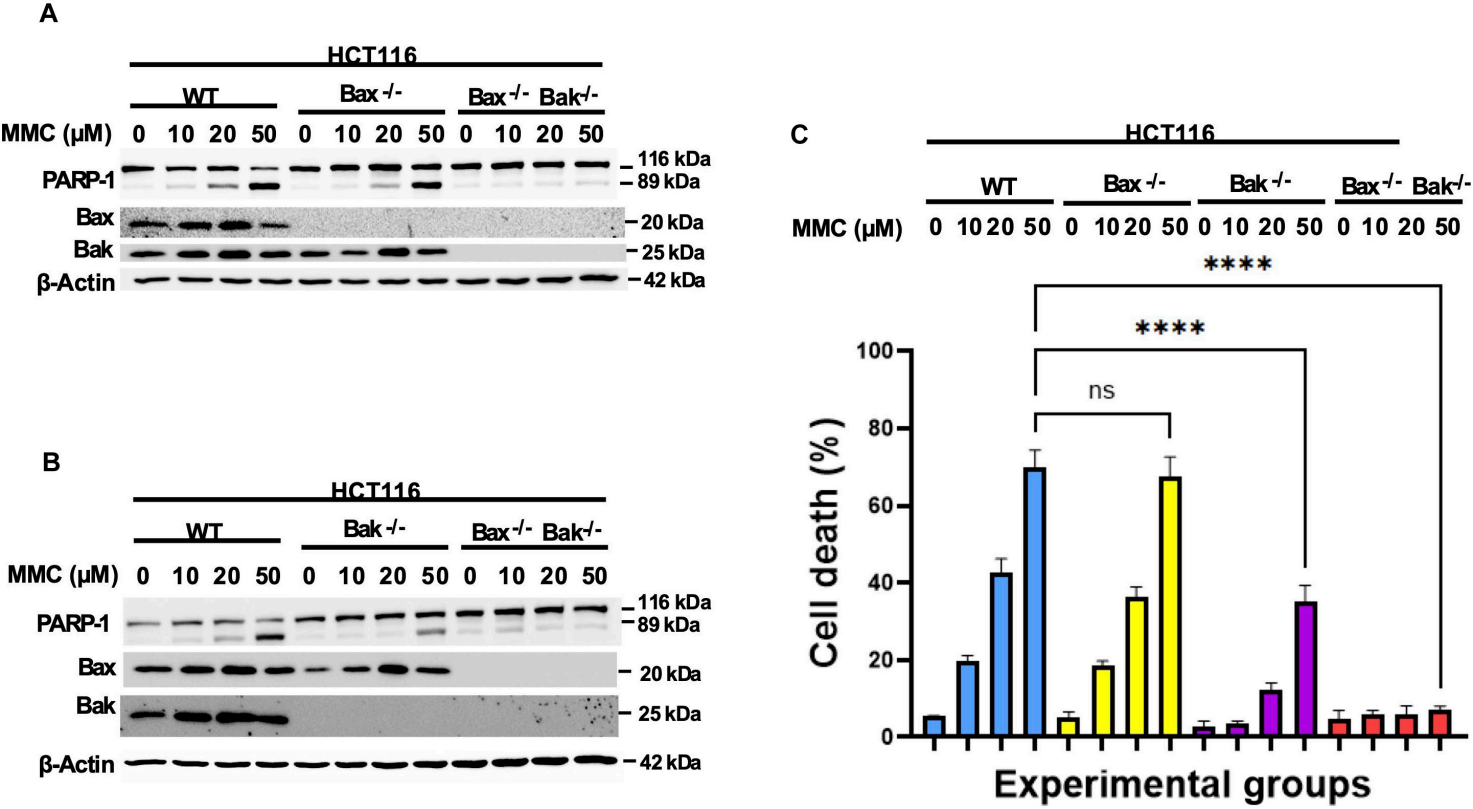
Role of Bax and Bak in chemotherapy-agent MMC-induced apoptosis. HCT116 WT, Bak^−/−^, Bax^−/−^, and Bak^−/−^/Bax^−/−^-DKO cells were treated with 10, 20, or 50 μM MMC for 24 h. (A) After treatment, whole-cell extracts were analyzed with an immunoblotting assay using indicated antibodies to compare treatment effects in WT HCT116 cells, Bax^−/−^ HCT116 cells, and Bak^−/−^/Bax^−/−^-DKO HCT116 cells. (B) Whole-cell extracts were analyzed with an immunoblotting assay using indicated antibodies to compare treatment effects in WT HCT116 cells, Bak ^−/−^ HCT116 cells, and Bak^−/−^/Bax^−/−^-DKO HCT116 cells. (C) After treatment, cell death was determined by a trypan blue exclusion assay. Error bars represent the mean + SD from triplicate experiments. ****P = 0.0001. ns, not significant.

As this study compared ART-EMs and TRAIL’s dependence on BH3 domain protein, Bid, with chemotherapeutic agent, MMC’s independence of Bid, differential reliance on Bak instead of Bax in chemotherapy agent-induced cytotoxicity is also observed [36]. MMC at a dosage of 50 μM, unlike the combinatorial treatment using ART-EMs and TRAIL, shows significant PARP-1 cleavage in Bax-deficient samples, which demonstrates that MMC-induced apoptosis is Bax-independent (Fig. 7A). Comparatively, data from Fig. 7B clearly reveal significant reduction of PARP-1 cleavage in Bak-deficient as well as Bax–Bak DKO samples treated with MMC. Cell viability assay confirmed that Bak-deficient, not Bax-deficient, samples demonstrate significant reduction in cell death during MMC treatment (Fig. 7C). Interestingly, Bax–Bak DKO cell lines treated with MMC show no PARP-1 cleavage (no apoptosis) (Fig. 7A and B) and no cytotoxicity (Fig. 7C). These results taken together indicate that MMC has a preferential dependence on Bak instead of Bax.

### MMC induces cytotoxicity independent of Bax and Bid, but dependent on p53 regulatory protein

**Fig. 8.**
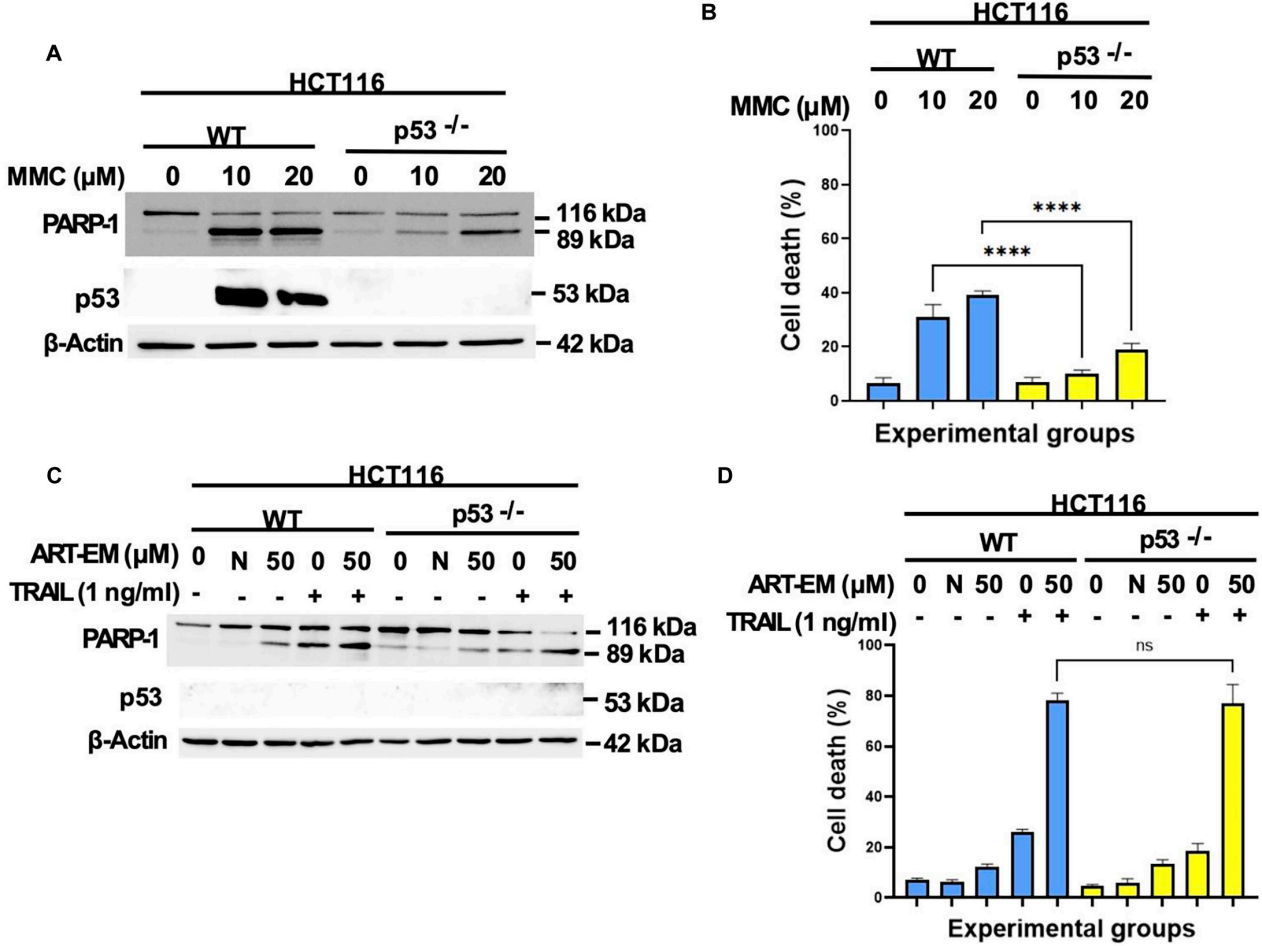
Comparison of the role of p53 in chemotherapy agent MMC-induced apoptosis versus ART-EM-enhanced TRAIL-induced apoptosis. HCT116 WT and p53^−/−^ cells were treated with 10 or 20 μM MMC for 24 h or 50 μM ART-EM for 20 h and then treated with or without 1 ng/ml rhTRAIL for another 4 h. (A and C) After treatment with MMC (A) or ART-EM ± rhTRAIL (C), whole-cell extracts were analyzed with an immunoblotting assay using indicated antibodies. (B and D) After treatment with MMC (B) or ART-EM ± rhTRAIL (D), cell death was determined by a trypan blue exclusion assay. Error bars represent the mean + SD from triplicate experiments. ****P = 0.0001. ns, not significant.

All these results emphasize the differential pathways of apoptosis between ART-EMs combined with TRAIL and chemotherapy agent MMC. Bid and Bax are key players in the synergy between ART-EMs and TRAIL’s enhancement of apoptosis in cancer cell lines. Results using MMC treatment suggest that MMC follows a different pathway to apoptosis. Previous research indicates that the transcription factor p53 responds to DNA damage induced by MMC treatment [27,29]. Results from immunoblot assay and trypan exclusion assay confirm MMC’s dependence on p53 as a regulatory protein in MMC-induced apoptosis (Fig. 8A) and cytotoxicity (Fig. 8B). There is reduced PARP-1 cleavage and cytotoxicity in p53-deficient cell lines compared to the WT control during treatment with MMC. The combinatorial treatment of ART-EMs and TRAIL was used in p53-deficient cell lines as a comparison (Fig. 8C and D). There is no significant reduction of PARP-1 cleavage and cytotoxicity in p53-deficient cells (Fig. 8C and D). Additionally, p53 is not detected in any of the samples treated with ART-EMs ± TRAIL, including the WT extracts (Fig. 8C). On the other hand, in MMC-treated samples, WT extracts reveal an increase in cellular levels of p53, emphasizing MMC’s reliance on p53 as a regulatory protein to apoptosis (Fig. 8A).

### Differential role of Bax/Bak in orchestration of cell death pathways

**Fig. 9.**
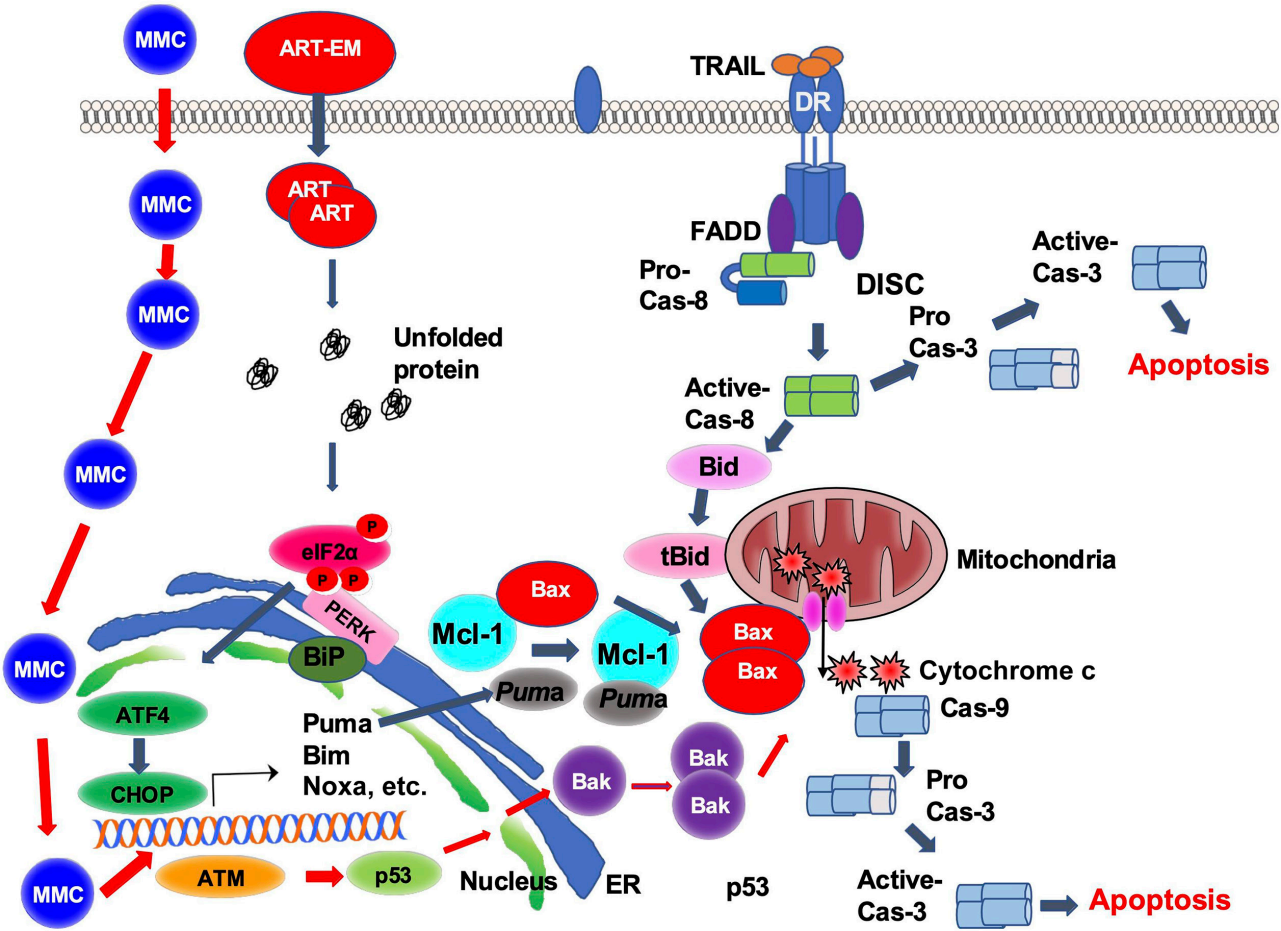
A schematic diagram of model for differential role of Bax/Bak in the orchestration of cell death pathways during treatment with ART-EM in combination with TRAIL or MMC alone.

Figure 9 shows a schematic diagram of model for the differential roles of Bax/Bak in the orchestration of cell death pathways during treatment with ART-EM in combination with TRAIL or MMC alone. The tumoricidal efficacy of AT (ART-EM + TRAIL) is associated with the Bid–Bax signal pathway. However, unlike AT treatment, the tumoricidal efficacy of MMC is dependent on the p53–Bak-associated pathway.

## Discussion

Our previous studies reveal that ferroptotic agents up-regulate DR5 gene expression, which enhances Bid cleavage through caspase-8 activation [37]. The synergistic apoptosis induced by the combined treatment of ferroptotic agents and TRAIL is inhibited in CHOP (C/EBP homologous protein)-deficient cells and DR5 knockdown cells, suggesting a critical role of the CHOP–DR5–Bid axis in the apoptosis process [13,34]. Bid, cleaved by caspase-8, translocates to the mitochondria and activates Bax through oligomerization during combined ART-EMs and TRAIL treatment [10,38]. tBid may also facilitate the opening of the mitochondrial permeability transition (PT) pore, resulting in cytochrome c release [39,40]. However, others have not found the importance of the PT pore in the cytochrome c-releasing activity of Bid [41]. To clarify this, we plan to investigate the role of Bid in cytochrome c release by using Bid-deficient (Bid KO), caspase-resistant mutant Bid D60E, and BH3-defective mutant Bid G94E HCT116 cells in future studies.

Our studies also demonstrate that the mitochondria-dependent caspase pathway plays an important role in ART-EM-enhanced TRAIL cytotoxicity. A fundamental question remains: How does ART-EMs promote TRAIL-induced cytochrome c release? In healthy cells, cytochrome c is localized in the mitochondrial intermembrane/intercristae spaces, where it functions as an electron shuttle. Apoptotic stimuli often cause outer membrane permeabilization, enabling cytochrome c mobilization from cardiolipin (CL) and subsequent release [40,42]. Future investigations into mitochondrial membrane potential alterations during combined ART-EMs and TRAIL treatment will help address the question of cytochrome c release.

Another critical aspect requiring further exploration is the stepwise activation and oligomerization of Bax and Bak, impacted by their structural differences, and how these factors influence the dependence of ART-EMs and TRAIL combinatorial treatment on Bax rather than Bak [43,44]. Bax is localized in the cytosol as a monomer, and its α1-helix undergoes activation and oligomerization via BH3 activation proteins tBid, Bim, or PUMA, exposing Bax’s N-terminal and BH3 domain, while disengaging the C-terminal α9-helix for mitochondrial targeting and membrane permeabilization [43,44]. In contrast, Bak remains continuously active in the MOM, with its C-terminal α9-helix exposed, and requires BH3 protein activation to oligomerize [43,44]. We hypothesize that oligomerized Bax, rather than Bak, forms the MOM pore responsible for cytochrome c efflux. However, previous studies suggest that Bax-induced cytochrome c release is independent of tBid [41], which warrants further clarification using triple KO Bax-, Bak-, and Bid-deficient cells. Additionally, the interactions between BH3 proteins (e.g., PUMA and tBid) and Bax versus Bak during ART-EMs + TRAIL treatment can be explored. Further studies on oligomerization and cytochrome c release will help elucidate the activation differences between Bax and Bak and the reasons for Bak’s lack of involvement in this specific apoptotic pathway.

Our results also highlight important differences between ART-EMs + TRAIL and traditional chemotherapeutic agents, such as MMC. As noted, ART-EMs and TRAIL-induced cytotoxicity is dependent on Bid and Bax (Figs. 3C and 6C), whereas MMC induces cytotoxicity independently of Bid (Fig. 5A and B). MMC-treated HCT116 cells, using Bim siRNA, suggest that MMC might be independent of BH3 domain protein involvement (Fig. 5D). Further investigations into the role of other BH3 domain proteins in MMC-induced apoptosis are needed. Additionally, we observed that MMC is more dependent on Bak than Bax (Fig. 7), contrasting with the Bax dependency observed in ART-EMs + TRAIL treatment. Notably, MMC induces p53-dependent apoptosis, whereas ART-EMs + TRAIL acts independently of p53 (Fig. 8). MMC is known to activate the DNA damage-dependent ATM-p53 pathway [27–29]. ATM detects DNA damage and, through autophosphorylation, activates p53, which in turn regulates ROS and glucose deprivation-induced ER stress signals [7,26,27]. Activated p53 induces apoptosis by regulating pro-apoptotic proteins such as PUMA and NOXA [45], which leads to Bak oligomerization. Based on our results showing Bak-dependent apoptosis in MMC-treated Bak-deficient cells (Fig. 7), we hypothesize that p53 may activate Bak rather than Bax, possibly due to p53’s competitive inhibition of Bax’s interaction with BH3 proteins (e.g., Bid/PUMA/Bim) [46]. This hypothesis is supported by studies suggesting that p53 and Bax share a binding pocket on BH3 activator proteins, which could hinder Bax’s oligomerization [10,46]. However, the BH3-Bak interaction is weaker, meaning that Bak’s activation by p53 might be less inhibited, explaining why Bak remains effective in MMC-induced apoptosis.

In the present study, we developed ART-EM and assess the tumoricidal efficacy of ART-EM in combination with TRAIL. The tumoricidal efficacy of the combined treatment may be promoted if TRAIL is encapsulated within the ART-EM due to preferential accumulation of microspheres in tumor regions. Co-encapsulation of TRAIL with ART within ART-EM could provide certain advantages, such as spatial and temporal co-delivery at the tumor site, potentially enhancing their synergistic effects. However, from a biological perspective, the pretreatment of ART (ferroptotic agent-induced ER stress response) is required for maximalized TRAIL-induced tumoricidal efficacy (apoptosis) [13]. Thus, we expect that TRAIL would be more effective when separate treatment is performed. Moreover, considering technical aspects, TRAIL is a protein with limited stability and thus requires distinct encapsulation strategies, such as a different polymer matrix or organic solvent, to maintain bioactivity during fabrication. Alternatively, we can develop a secretory TRAIL-armed natural killer (NK) cell-based therapy in combination with ART-EM. We previously reported that secretory TRAIL-armed NK cells are able to accumulate selectively at tumor sites and exert tumoricidal effects through sustained TRAIL release [47]. Obviously, further studies are necessary to develop these approaches in the near future.

In conclusion, the combination of ART-EMs with TRAIL holds promise as a second-line therapeutic approach, enhancing the efficacy of tumor-targeting therapies like HIAI, TACE, and chemotherapeutics. The synergistic apoptotic effect of ART-EMs + TRAIL relies on the crosstalk between ferroptosis-induced ER stress and the TRAIL-induced intrinsic Bid–Bax mitochondrial apoptosis pathway. This combinatorial treatment increases caspase activation and PARP-1 cleavage, with Bid and Bax playing pivotal roles in the apoptotic process. In contrast, MMC operates through a different mechanism, independent of Bid, Bim, and Bax, and relies on Bak for its cytotoxic effects. However, key questions remain regarding the differential roles of Bax and Bak in the Bid–Bax pathway versus the p53–Bak pathway during ART-EMs + TRAIL versus MMC treatments, which will be explored in future studies.

## Data Availability

Research materials that were generated in the studies including plasmid DNA constructs will be made freely available to the scientific research community as soon as this manuscript has been documented in a publication. Raw data were generated at the Cedars-Sinai Medical Center. Derived data supporting the findings of this study are available from the corresponding author (Y.J.L.) on request.
